# Extensive Sympatry and Frequent Hybridization of Ecologically Divergent Aquatic Plants on the Qinghai-Tibetan Plateau

**DOI:** 10.3389/fpls.2022.851151

**Published:** 2022-05-12

**Authors:** Zhigang Wu, Zhong Wang, Dong Xie, Juan Zhang, Pengsen Cai, Xing Li, Xinwei Xu, Tao Li, Jindong Zhao

**Affiliations:** ^1^State Key Laboratory of Freshwater Ecology and Biotechnology, Institute of Hydrobiology, Chinese Academy of Sciences, Wuhan, China; ^2^Department of Ecology, College of Life Science, Wuhan University, Wuhan, China; ^3^Co-Innovation Center for Sustainable Forestry in Southern China, College of Biology and the Environment, Nanjing Forestry University, Nanjing, China; ^4^National Wetland Ecosystem Field Station of Taihu Lake, National Forestry Administration, Suzhou, China; ^5^State Key Laboratory of Protein and Plant Genetic Engineering, College of Life Sciences, Peking University, Beijing, China

**Keywords:** altitudinal gradient, hybrid zone, hydrophilous plant, introgression, population genetics, niche similarity, species distribution model

## Abstract

Hybridization has fascinated biologists in recent centuries for its evolutionary importance, especially in plants. Hybrid zones are commonly located in regions across environmental gradients due to more opportunities to contact and ecological heterogeneity. For aquatic taxa, intrazonal character makes broad overlapping regions in intermediate environments between related species. However, we have limited information on the hybridization pattern of aquatic taxa in alpines, especially submerged macrophytes. In this study, we aimed to test the hypotheses that niche overlap and hybridization might be extensive in related aquatic plants across an altitudinal gradient. We evaluated the niche overlap in three related species pairs on the Qinghai-Tibetan Plateau and assessed the spatial pattern of hybrid populations. Obvious niche overlap and common hybridization were revealed in all three pairs of related aquatic plants. The plateau edge and river basins were broad areas for the sympatry of divergent taxa, where a large proportion of hybrid populations occurred. Hybrids are also discretely distributed in diverse habitats on the plateau. Differences in the extent of niche overlap, genetic incompatibility and phylogeographic history might lead to variation differences in hybridization patterns among the three species pairs. Our results suggested that plateau areas are a hotspot for ecologically divergent aquatic species to contact and mate and implied that hybridization may be important for the freshwater biodiversity of highlands.

## Introduction

Hybridization between related species or genetic lineages is a common phenomenon in nature. Generally, allopatric or ecologically isolated taxa with weak reproductive barrier contact and mate in limited regions of overlap, forming hybrid zones (Barton and Hewitt, 1985; Seehausen, 2004). Ecologists have regarded hybrid zones as natural laboratories in which to study evolutionary processes because hybridization provides a great contribution to the accumulation of genetic variation, the generation of novelty, and speciation, especially in plants (Barton and Hewitt, 1985; Arnold, 1997; Taylor and Larson, 2019). Swarms in hybrid zones could also be useful resources to explore adaptive traits from the perspective of genetic conservation and landscape management (Hamilton and Miller, 2016; Matthews et al., 2020).

For related species with ecological divergence, parents are typically located at opposite extremes, whereas their ranges can overlap in intermediate environments (Barton, 2001). As reviewed in Abbott (2017), a large proportion of hybrid zones occur across environmental gradients, which provide a favorable niche for both parental species. The structure and types of hybrid zones in these areas vary, depending on the fitness of hybrids to their parents in heterogeneous niches (Barton and Hewitt, 1985; Harrison, 1986; Abbott, 2017). Hence, various habitats may facilitate the contact of related species and the formation of hybrid populations (Yakimowski and Rieseberg, 2014). Exploring natural hybrid zones and studying their patterns in regions across environmental gradients are also hotspots in the research field of biodiversity.

Highlands are particularly good systems for examining interactions and interspecies gene flow between related species because steep changes in ecological variables facilitate the coexistence of taxa with different distribution characteristics (Abbott and Brennan, 2014). They may also serve as marginal or new areas for parental plants, and hybrids may have more opportunities to persist when benefiting from adaptation to unique environments and limited competition (Arnold, 1997; Barton, 2001; Arnold and Martin, 2010). According to relatively few case studies, hybrid zones across altitudinal gradients encompass all types of hybrid zone models and have the potential to contain high levels of phenotypic, genetic, and adaptative variation (Arnold, 1997; Abbott and Brennan, 2014; Wu et al., 2022).

In aquatic taxa, hybridization is also an important mechanism for the maintenance and amplification of variation (Barrett et al., 1993; Les and Philbrick, 1993). Many widespread aquatic macrophytes have closely related species with boreal distributions, and hybrids could be found in areas of their northern ranges (Santamaría, 2002). Diverse aquatic plants also occur in alpine regions (Wang, 2003). Considering the intrazonal character of aquatic flora, plateaus and their adjacent areas are expected to provide potential regions for the overlaps of ecologically divergent species as well, which may subsequently influence aquatic biodiversity regionally. However, we know little about the niche overlap of related aquatic plants in alpine ecosystems and its consequences on the spatial pattern of genetic variation. Here, we aimed to validate the hypotheses that (1) the niche overlap might be extensive in related aquatic plants in highland regions and (2) hybridization events were frequent, and the distribution of hybrid populations was related to the spatial pattern of ecologically divergent plants.

Our research was performed on the Qinghai-Tibetan Plateau (QTP). The QTP is the highest plateau in the world and an ideal region for research on hybridization because it provides a complex landscape and novel environments in which plants can persist, which is driven by elevational gradients (Shen et al., 2014; Sun H. et al., 2014). As the origin of many great rivers in Asia, the QTP is full of wetlands, which provide abundant habitats for aquatic life (Wang and Dou, 1998). To test the hypotheses, we chose three typical species pairs of submerged plants with ecological divergence. Hybridization between all species pairs has been reported previously (Hollingsworth et al., 1996; Moody and Les, 2002; Wiegleb et al., 2017). Based on previous studies and our initial work, they cooccurred on the QTP with different distribution patterns (Wang, 2003; Wu et al., 2015; Du and Wang, 2016). In the present study, we conducted an overall survey on the distribution of the three species pairs. We quantified the ecological niche and potential distribution of each species and measured the overlap between related plants. Molecular markers were used to complement the identification of their hybrid. We also conducted comparisons of the niches between hybrid populations and their parents and discussed the differences in hybridization patterns among the three species pairs.

## Materials and Methods

### Species

#### *Myriophyllum spicatum* and *Myriophyllum sibiricum*

*Myriophyllum spicatum*, also called Eurasian watermilfoil, is one of most important alien weeds in the world. The species is native to Eurasia and northern Africa and invaded North America in the 19th century (Aiken, 1981; Couch and Nelson, 1985). Now, it occurs on all continents except Antarctica. It is distributed in cold regions north to 60°N. Its sister species *Myriophyllum sibiricum* is native to Eurasia and North America and presents a circumboreal distribution, rarely extending south of the mean isotherm of 0°C in winter (Aiken, 1981; Ceska and Ceska, 1986; Moody and Les, 2002). Hybridization between the species has been reported in North America (Moody and Les, 2002, 2007) and East Asia (Wu et al., 2015). Morphologically, the two species can be distinguished by leaf segment numbers and lengths.

#### *Stuckenia pectinata* and *Stuckenia filiformis*

*Stuckenia pectinata* is a perennial submerged aquatic macrophyte with a cosmopolitan distribution that occurs on all continents except Antarctica and does not reside in polar regions (Kaplan, 2008). It occurs in a wide range of habitats, most often in lentic wetlands but also in running water. It tolerates brackish water (up to 20‰ salinity) and may grow in arid zones, on high mountains or at seasides (Nies and Reusch, 2005; Kaplan, 2008; Li et al., 2015). *Stuckenia filiformis* is widespread in the Northern Hemisphere and common in Siberia and the mountain regions of Central Asia (Kaplan, 2008). *Stuckenia filiformis* tends to grow in cold and shallow water and is also able to tolerate saline habitats (Kaplan, 2008). Both *Stuckenia* species are extremely variable in morphology and distinguishable by the structure of leaf sheaths, branching pattern, and fruit size (Hollingsworth et al., 1996). Hybrids between the two species have been identified in the northern half of Europe, Siberia, and China (Hollingsworth et al., 1996; Kaplan, 2008; Du and Wang, 2016) and named *S.* x *suecicus*.

#### *Ranunculus trichophyllus* and *Ranunculus subrigidus*

*Ranunculus trichophyllus* is a perennial aquatic plant with a subcosmopolitan distribution. The species is morphologically and genetically polymorphic. The plant grows in various habitats, particularly in alkaline waters. The morphological overlap and hybridization of *R. trichophyllus* with many other *R.* section *Batrachium* species has been reported (Zalewska-Gałosz et al., 2015; Wiegleb et al., 2017). *Ranunculus subrigidus* is an Amphipacific species with an arid to subarctic distribution and tends to colonize inland habitats with hard or even brackish water. It is separated from *R. trichophyllus* by rigid leaves, length of adjacent internodes and flower size (Wiegleb et al., 2017). Hybridization between the two species occurred but has not been well-studied.

### Field Work

**FIGURE 1:**
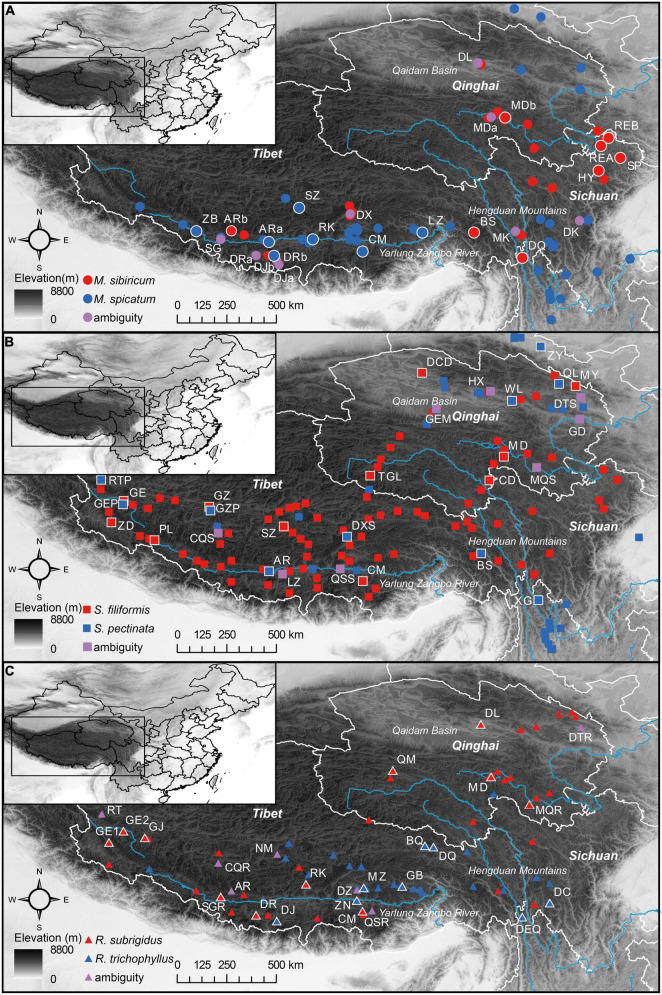
Geographic distribution of each parental species [**(A)** *Myriophyllum*, **(B)** *Stuckenia*, and **(C)** *Ranunculus*] and morphologically ambiguous populations on the Qinghai-Tibetan Plateau. The pure populations used in molecular identification are labeled and outlined in white.

Field surveys on the QTP were performed from 2010 to 2015, and in total, 254 sites were examined (Supplementary Figure 1). Based on morphological identification, *M. spicatum* and *M. sibiricum* were found at 46 and 28 sites, *S. pectinata* and *S. filiformis* were found at 40 and 92 sites, and *R. trichophyllus* and *R. subrigidus* were found at 35 and 54 sites (Figure 1). In the summer of 2015, our field work was focused on mixed populations and plant material collection for molecular analysis. We did not detect new mixed *Myriophyllum* populations, and a total of 25 sites (*Myriophyllum*: 10 sites that have been included in our previous work, Wu et al., 2015; *Stuckenia*: 8 sites; *Ranunculus*: 7 sites) contained individuals of both parental species or individuals with morphological ambiguity (Figure 1 and Table 1). The plant materials were also sampled and preserved in silica gel for the molecular identification of hybrids.

**TABLE 1:** Geographic locations, numbers of samples and hybrids, Q values of STRUCTURE and genotype class assignment of NewHybrids revealed by microsatellite markers, and cpDNA haplotypes for the populations involved in hybridization events.

Species	Code	Loc	Coor	Alt	NI	NH	Q	GC	Hap
*Myriophyllum*	SG	Saga, Tibet	29.32°N, 85.23°E	4701	15	15	0.640–0.690	F1	A3
*Myriophyllum*	ARb	Angren, Tibet	29.68°N, 85.72°E	5111	20	1	0.569	F2	B4
*Myriophyllum*	DRa	Dingri, Tibet	28.59°N, 86.83°E	4314	19	19	0.007–0.009	BW	B4, B5
*Myriophyllum*	DJa	Dingjie, Tibet	28.36°N, 87.76°E	4208	15	15	0.004–0.041	BW	B5
*Myriophyllum*	DJb	Dingjie, Tibet	28.17°N, 87.87°E	4253	12	12	0.006–0.007	BW	B5
*Myriophyllum*	DX	Dangxiong, Tibet	30.47°N, 91.10°E	4270	20	20	0.198–0.461	F1	B1
*Myriophyllum*	MK	Mangkang, Tibet	29.67°N, 98.59°E	3878	7	6	0.612–0.842	F1, BW	B8, B9
*Myriophyllum*	MDa	Maduo, Qinghai	34.84°N, 98.13°E	4200	30	6	0.654–0.694	F2, BB	A1
*Myriophyllum*	DL	Delingha, Qinghai	37.31°N, 96.90°E	2817	13	13	0.667–0.697	F1	B1, B6
*Myriophyllum*	KD	Kangding, Sichuan	30.16°N, 101.49°E	3403	13	13	0.594–0.863	F1, F2, BB, BW	B6
*Stuckenia*	DTS	Datong, Qinghai	37.1°N, 101.57°E	2628	3	1	0.376	F2	1
*Stuckenia*	GD	Guide, Qinghai	36.11°N, 101.52°E	2194	19	5	0.256–0.483	F2	22
*Stuckenia*	HX	Haixi, Qinghai	37.37°N, 97.47°E	3045	12	1	0.610	F2	1
*Stuckenia*	GEM	Geermu, Qinghai	36.59°N, 95.01°E	2755	7	1	0.657	NI	2
*Stuckenia*	MQS	Maqin, Qinghai	34.37°N, 100.25°E	3843	15	5	0.300–0.860	F2	1, 2, 20
*Stuckenia*	CQS	Cuoqin, Tibet	30.94°N, 85.12°E	4676	16	6	0.200–0.827	F2	18
*Stuckenia*	LZ	Lazi, Tibet	29.09°N, 88.04°E	4342	10	3	0.314–0.473	F2	18
*Stuckenia*	DXR	Dangxiong, Tibet	30.78°N, 90.96°E	4699	8	1	0.164	F2	18
*Ranunculus*	DTR	Datong, Qinghai	37.1°N, 101.57°E	2628	11	2	0.647–0.803	F1	S2
*Ranunculus*	MQR	Maqin, Qinghai	33.90°N, 99.55°E	4036	14	1	0.757	BB	S2
*Ranunculus*	RT	Ritu, Tibet	33.17°N, 79.84°E	4331	16	3	0.106–0.219	F1	T3
*Ranunculus*	CQR	Cuoqin, Tibet	30.94°N, 85.12°E	4676	14	3	0.118–0.360	F1	T1
*Ranunculus*	AR	Angren, Tibet	29.69°N, 85.72°E	5111	21	3	0.112–0.180	F1	T1
*Ranunculus*	NM	Nima, Tibet	31.35°N, 87.8°E	4656	12	7	0.152–0.303	F1	T1
*Ranunculus*	DJ	Dingjie, Tibet	28.32°N, 87.77°E	4208	16	3	0.005–0.022	F1	T1
*Ranunculus*	DZ	Dazi, Tibet	29.72°N, 91.41°E	3613	12	2	0.230–0.253	F1	T1
*Ranunculus*	QSR	Qushui, Tibet	28.78°N, 92.09°E	4490	26	7	0.118–0.478	F1	T1

*Loc, Location; Coor, Coordinate; Alt, Altitude; NI, Number of individual; NH, Number of hybrid individuals; Q, Q-values of hybrid individuals generated in STRUCTURE, close to 0 or 1 represent purebred widespread parental species (M. spicatum, S. pectinate, and R. trichophyllus) or boreal parental species (M. sibiricum, S. filiformis, and R. subrigidus), respectively; GC, hybrid assignment to five Genotype Classes [F1, F2, backcross-widespread parental species (BW), backcross-boreal parental species (BB), and non-identified(NI)], suggested by NewHybrids; Hap, haplotype of hybrid individuals, M. spicatum: A1–A5, M. sibiricum: B1–B9, S. pectinata: 22–24, S. filiformis: 1–21, R. trichophyllus: T1–T4, R. subrigidus: S1–S2.*

### Niche Analysis

The variables climate, soil, and landcover were used as environmental factors for the niche analysis (Table 2). Environmental data for the entire studied region (25–40°N, 75–105°E) were obtained from open sources with a resolution of 1 km (climate: www.worldclim.org; soil: Harmonized World Soil Database, v 1.2; landcover: www.resdc.cn; Table 2). Because autocorrelated variables may bias the niche comparison analyses (Broennimann et al., 2012), we kept one of any set of highly correlated variables (Pearson’s *r* > 0.75). Thirteen variables were selected as evaluator variables, including five bioclimatic variables (Bio1, Bio2, Bio3, Bio4, and Bio12), altitude, solar radiation, average wind speed in the growing season, three soil parameters (texture type, pH, and salinity), and landcover (Table 2).

**TABLE 2:** Ecological variables and their relative percentage contributions in the Maxent models.

Code	Variable	MSp	MSi	SP	SF	RT	RS
bio1	Annual temperature	13.21	27.19	7.24	14.72	7.69	23.32
bio2	Mean diurnal range	3.29	1.10	8.49	3.21	1.52	1.69
bio3	Isothermality	13.64	0.20	10.91	3.35	21.62	2.96
bio4	Temperature seasonality	6.15	0.00	1.46	5.52	5.54	0.81
bio12	Annual precipitation	13.14	13.42	2.62	14.66	8.61	9.61
dem	Elevation	16.53	8.57	24.22	36.41	30.82	10.64
srad	Solar radiation	8.93	0.00	7.42	8.56	6.00	14.97
wind	Wind speed	0.33	0.02	2.48	1.85	2.69	0.02
tex	Topsoil USDA texture classification	6.57	20.38	7.14	2.99	14.90	3.86
ph	Topsoil pH	1.17	11.45	11.68	0.16	0.16	6.59
esp	Topsoil salinity	10.14	1.80	8.04	2.29	0.44	0.89
cov	Landcover	6.90	15.86	8.29	6.30	0.00	24.64

*MSp, M. spicatum; MSi, M. sibiricum; SP, S. pectinata; SF, S. filiformis; RT, R. trichophyllus; RS, R. subrigidus.*

The ecological niche of each plant was quantified through both a species distribution model (SDM) and an ordination approach. 1. The most widely used SDM algorithm, maximum entropy (Maxent, Phillips et al., 2006), was chosen to model the potential distribution of each species based on the locations of field-observed occurrence. Through initial runs, Maxent performed better on our dataset than other generally used algorithms, such as generalized additive models, boosted regression trees and random forest, based on the AUC (area under the receiver operator curve) and TSS (maximizing true positive rate/sensitivity and true negative rate/specificity) values. One-fifth of the presence was used as testing data for cross-validation, and the threshold of occurrence was generated based on the sum of the sensitivity and specificity. The algorithm either runs 1,000 iterations or ends at convergence (threshold = 0.00001). 2. An ordination approach, such as principal components analysis (PCA), was used to identify the niches of species observations, with the available ecological data of the entire study area as the background (Broennimann et al., 2012). The observed occurrences were converted into smooth densities and represented using the first two PC axes, which was named the “pca-env” technique by Broennimann et al. (2012). It was proven to perform well in the subsequent niche overlap analysis among different ordination and niche model techniques (Broennimann et al., 2012). When executing the analysis, we used the proportions of sand and clay instead of the categorical variable soil texture.

The niche overlap of each species pair was measured with D metrics (Warren et al., 2008; Broennimann et al., 2012). Statistical tests of niche equivalency were performed for the null hypothesis that the niches of the parental species were identical in the study area. The overlaps of the pseudoreplicate model were estimated by randomly reassigning the presence sites to a pair of species and then compared to the observed niche overlap (Warren et al., 2008). We also performed background/niche similarity tests for the null hypothesis that the empirical overlap between two species is greater than the simulated overlap between the niche of one species and the backgrounds of the other (Warren et al., 2008). Contributor variables of PCA were used for canonical discriminants analysis (CDA), to compare the niche requirements among parent species and their hybrids. Wilks’ test was employed to evaluate the goodness of fit statistic and measure the model performance.

All the above analyses were conducted in R version 3.6.2. The SDM-based analyses were performed using the packages “dismo” (Hijmans et al., 2017) and “ENMTools” (Warren et al., 2010). The Java program Maxent v 3.3.3k was also implemented in “dismo” (Hijmans et al., 2017). The PCA-based analyses were performed in the package “ecospat” (Di Cola et al., 2017). The “candisc” package was used for CDA analyses (Friendly and Fox, 2021).

### Molecular Identification of Hybrids

Our previous work identified hybrids in mixed *Myriophyllum* populations using microsatellite and cpDNA markers (Wu et al., 2015). Molecular studies have also been suggested for the identification of *Stuckenia* and *Ranunculus* hybrids because they are morphologically variable and difficult to distinguish from the parental species (Kaplan, 2008; Zalewska-Gałosz et al., 2015). We conducted a molecular analysis of a total of 303 individuals from all 15 indeterminate populations for the two species pairs (*Stuckenia*: 91 individuals of 8 populations; *Ranunculus*: 112 individuals of 7 populations) (Figure 1 and Table 1). We also included parental populations of each species pair in the molecular analysis as reference data (Figure 1). A total of 21 pure *Stuckenia* populations (75 individuals of *S. pectinata* and 131 individuals of *S. filiformis*) and 20 pure *Ranunculus* populations (110 individuals of *R. trichophyllus* and 215 individuals of *R. subrigidus*) were involved.

Total genomic DNA was extracted using the DNAsecure Plant Kit (Tiangen Biotech, Beijing, China). Microsatellite markers (7 loci for Stuckenia, Nies and Reusch, 2004; Wu et al., 2020; 17 loci for Ranunculus, Wu et al., 2017; Wu et al., 2019) and one cpDNA fragment (*rpl32-trnL*) were chosen to identify kinships. The primers “rpL32-F” and “trnL^(UAG)^” (Shaw et al., 2007) were used to amplify and sequence the region. The PCR conditions were performed according to the respective protocols.

Microsatellite genotyping was performed using GeneMarker v1.5 (SoftGenetics, State College, PA, United States). A Bayesian clustering method approach was used for individual genetic assignment, implemented in STRUCTURE v2.3.4 (Pritchard et al., 2000; Falush et al., 2003). Ten independent runs were performed at *K* = 2 with a burn-in period of 500,000 iterations and 2,000,000 MCMC iterations under the admixture model. The average Q value of all STRUCTURE runs was used to indicate individuals as parental species or hybrids, using Q < 0.10 or Q > 0.90 for pure individuals and 0.10 < Q < 0.90 for hybrids (Burgarella et al., 2009). We also assessed the optimal number of clusters for further genetic divergence (Evanno et al., 2005), when *K* = 2 was suggested for all species pairs.

To mutually verify hybrid identification and evaluate hybrid class assignment, a model-based Bayesian approach was applied using NewHybrids to further assign all individuals to one of six possible classes through evaluation of the Bayesian posterior probability of membership: two parental species, F1s, F2s, and backcrosses to both parents (Anderson and Thompson, 2002). In NewHybrids program, pure samples of the two species need not be specified for hybrids identification, because the allele frequencies of the two species are not required as *a priori*. The runs were performed using Jeffreys’ prior distribution, with a burn-in of 200,000 iterations and a run length of 2,000,000 iterations. The threshold of assignment probability was set to 75%. When *Myriophyllum* populations were analyzed, the same dataset was used as that in Wu et al. (2015). All cpDNA fragment sequences were aligned using the program Mafft v6.7 (Katoh and Toh, 2008). The cpDNA haplotypes were extracted using DNASP v5.10 (Librado and Rozas, 2009). The published *rpl32-trnL* sequences for each genus (for *Ranunculus*, *R.* section *Batrachium* species were included) were used to confirm that no other closely related species involved. We implemented a median-joining network for the genealogical relationships among the genotypes and haplotypes in NETWORK v10.1 (Bandelt et al., 1999).

## Results

### Potential Distributions and Niche Overlaps Based on the Species Distribution Model

The potential distribution of each species was quantified using Maxent. The AUC of the training values ranged from 0.935 to 0.957 for the SDM of the six parental species (Supplementary Table 1), indicating that the models of all species performed well and generated excellent evaluations. The thresholds of presence/absence for each species ranged from 0.211 to 0.276, as suggested by the TSS (Supplementary Table 1). We defined and represented the species potential habitats as “high potential” (>0.6), “moderate potential” (0.4–0.6), “low potential” (threshold-0.4) and “no potential” (<threshold), based on the probability of presence suggested by the models (Supplementary Figure 2).

Temperature, precipitation, and elevation were the most decisive environmental factors for the distribution of *M. spicatum* on the QTP (Table 2). Its highly suitable region on the QTP was found to include the edge of the plateau and the river valley areas (Supplementary Figure 2A). The distribution of *M. sibiricum* can be mainly attributed to the conditions of temperature and soil texture. The northern slope of the Himalayas and border areas among Qinghai, Sichuan, and Tibet were the main regions that were suitable for *M. sibiricum* (Supplementary Figure 2B). Elevation, temperature isothermality, and soil pH were the environmental variables that constrained the distribution of *S. pectinata* (Table 2). Its suitable areas were mainly at the northeast and southeast edges of the QTP (Supplementary Figure 2C). Elevation, annual temperature, and precipitation mainly influenced the distribution of *S. filiformis* (Table 2). The species had broad potential distribution areas over the plateau (Supplementary Figure 2D). Elevation, temperature isothermality, and soil texture were the main factors influencing the distribution of *R. trichophyllus* (Table 2), which was most likely to occur in central Tibet and western Sichuan (Supplementary Figure 2E). Annual temperature, landcover, and solar radiation greatly affected the distribution of *S. subrigidus* (Table 2). It was the second most common submerged plant on the QTP and had extensive suitable distribution areas (Supplementary Figure 2F).

**FIGURE 2:**
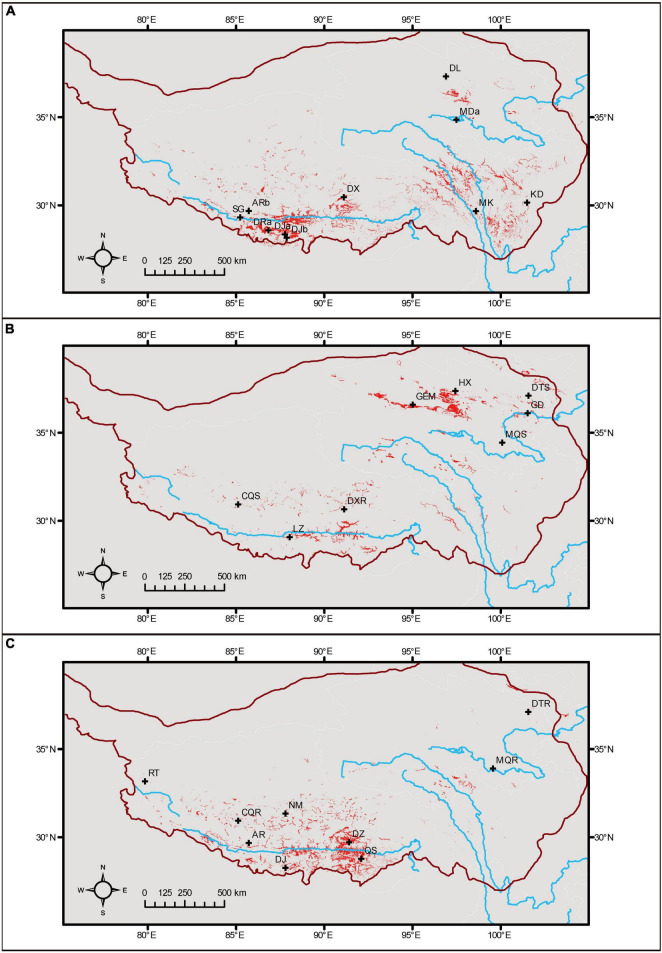
Potential coexistence regions for related species, based on the presence in species distribution models. **(A)** *Myriophyllum*, **(B)** *Stuckenia*, and **(C)** *Ranunculus*. Sites of hybrid populations were plotted, and the boundary of the QTP is shown.

By multiplying the presence distributions (>threshold) of related species, we obtained the potential areas for the coexistence of each species pair (Figure 2). For *Myriophyllum*, the two species could be separated by the conditions of temperature, seasonality, elevation, and solar radiation (Supplementary Figure 3). The potential sympatric areas for the two species were river valleys in southern Tibet and western Sichuan (Figure 2A). For *Stuckenia*, the temperature diurnal range and seasonality, elevation, wind speed, and soil texture were decisive for the coexistence of the two species (Supplementary Figure 4). Their potential sympatric regions were medium-elevation areas in north Qinghai and mosaic areas along the Yarlung Tsangpo River (Figure 2B). The annual temperature and elevation mainly separated the two *Ranunculus* species on the QTP (Supplementary Figure 5). They might cooccur in central and southern Tibet (Figure 2C).

Obvious niche overlap was suggested in all species pairs (*Myriophyllum D* = 0.594; *Stuckenia D* = 0.424; *Ranunculus D* = 0.545, Table 3). The null hypothesis of niche equivalency was rejected in all species pairs, indicating that the niches of ecologically isolated parental species did not shift to equivalence on the QTP (Table 3 and Supplementary Figure 6). The results of the background test in *Myriophyllum* and *Ranunculus* were significant, indicating that the niche overlap between the two parental species pairs was more obvious than that between either parent and random background points (Table 3 and Supplementary Figure 7).

**TABLE 3:** Niche overlap index and significance in tests of niche equivalency and backgrounds for related species pairs.

	SDM			PCA		
	D	Sig. of NIT	Sig. of BT	D	Sig. of NIT	Sig. of BT
*Myriophyllum*	0.594	NS	*/**	0.625	NS	*/*
*Stuckenia*	0.424	NS	NS/NS	0.446	NS	NS/NS
*Ranunculus*	0.545	NS	**/*	0.516	NS	*/*

NIT, niche equivalency test; BT, background tests between widespread/boreal parent species and random points in backgrounds; NS, not significant; *p < 0.05; **p < 0.01.

### Niche Overlap Based on the Ordination Approach

The ecological niche was also measured by an ordination approach. The first two axes in PCA explained 76.61% of the variation in environmental factors (Supplementary Figure 8). Axis 1 was associated with the temperature variables, precipitation, soil texture, and wind speed, while axis 2 was mainly associated with the elevation and solar radiation (Supplementary Figure 8).

**FIGURE 3:**
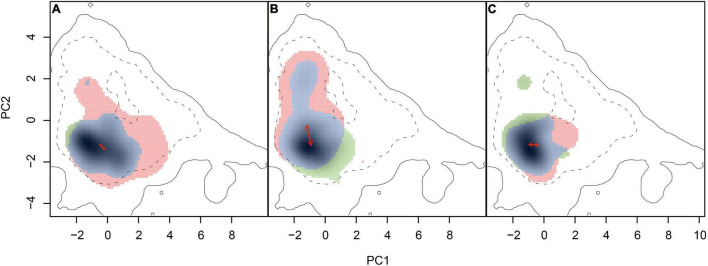
Niche overlap between related species. **(A)** *Myriophyllum*, **(B)** *Stuckenia*, and **(C)** *Ranunculus*. The solid and dashed contour lines indicate 100 and 50% of the available environmental data for the whole study region as backgrounds. The unique niches of widespread species (red: *M. spicatum*, *S. pectinate*, and *R. trichophyllus*), the unique niches of boreal species (green: *M. sibiricum*, *S. filiformis*, and *R. subrigidus*), and the overlap (blue) were represented, respectively. The solid arrows represent the differences in the niche density center between parental species.

Obvious niche overlap was also identified when quantified using the ordination approach (Figure 3). Limited unique niches for each parental species and few changes in niche centers were found for *Myriophyllum* and *Ranunculus* (Figures 3A,C). The niche overlap between *S. pectinata* and *S. filiformis* was obvious as well, while the high-occurrence density areas of the two species were different (Figure 3B). The values of niche overlap between each species pair were 0.625 (*Myriophyllum*), 0.446 (*Stuckenia*), and 0.516 (*Ranunculus*) (Table 3 and Figure 3).

Similar to the results based on SDM, no niches of species pairs were identical according to the niche equivalency tests (Table 3 and Supplementary Figure 9). The background tests showed that the niche similarity between *Myriophyllum* species and between *Ranunculus* species was higher than expected at random on the QTP (Table 3 and Supplementary Figure 10). Consistent with the results of background tests, CDA analyses yielded one discriminant function that explained 100% of the variance in PC contributors, showing ecological niche differences were significant between parent *Stuckenia* (Wilks’ λ = 0.669, *p* < 0.001), but not significant between parental *Myriophyllum* species (Wilks’ λ = 0.705, *p* = 0.054) or *Ranunculus* species (Wilks’ λ = 0.774, *p* = 0.053) (Supplementary Figure 11). While moderate Wilks’ λ values (close to 1) suggested all species pairs occupied some regions that were ecologically similar.

### Molecular Identification of the Ambiguous Populations

Totals of 82 and 126 multilocus genotypes were revealed in the ambiguous populations of *Stuckenia* and *Ranunculus* using microsatellite markers. Under the assumption that *K* = 2, the two genetic clusters suggested by STRUCTURE analysis corresponded to the parental species (Supplementary Figure 12). All the individuals from parental populations had high posterior probabilities close to 0 and 1, except for DXS of *S. pectinata* and MQR of *R. subrigidus* (Supplementary Figure 12). Of the eight ambiguous *Stuckenia* populations, most contained individuals who were genetically admixed, with a probability range from 0.200 to 0.860 (Table 1, Figure 1B, and Supplementary Figure 12A), while QS was composed of two pure parental species. Of the seven ambiguous *Ranunculus* populations, all contained hybrids, with a probability range from 0.106 to 0.803 (Table 1, Figure 1C, and Supplementary Figure 12B). Two populations (DXS and MQR) assigned to *S. pectinata* and *R. subrigidus* by morphology included hybrid individuals (Table 1, Figure 1C, and Supplementary Figure 12B). The PL of *S. filiformis* contained one individual of *S. pectinata*.

**FIGURE 4:**
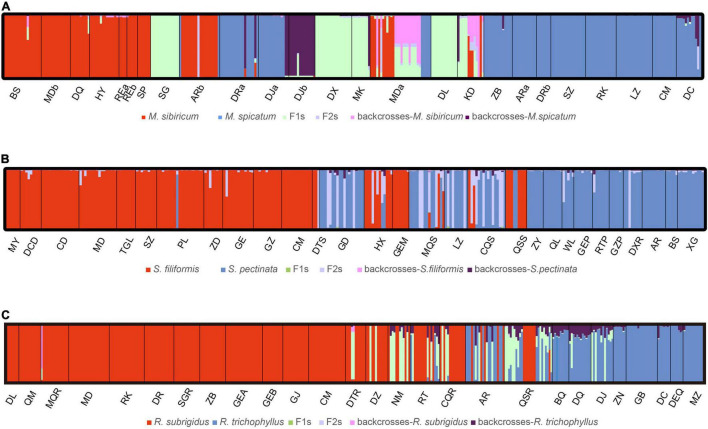
The results of the NewHybrids analysis for all species pairs based on microsatellite data. **(A)** *Myriophyllum*, **(B)** *Stuckenia*, and **(C)** *Ranunculus*. The vertical bars display the probability of assignment to six membership classes: two parents, F1s, F2s, and backcrosses to each parent.

The hybrid individuals suggested by NewHybrids were consistent with the results of STRUCTURE with few exceptions (Table 1 and Figure 4). All genotype classes were found in the *Myriophyllum* hybrids. In MDa, more hybrids were suggested compared to the STRUCTURE results (Table 1 and Figure 4A). *Stuckenia* hybrids were rarely F1s. The hybrid individuals in GEM suggested by STRUCTURE were assigned to *S. filiformis* (Table 1 and Figure 4B). We assessed the STRUCTURE results under *K* = 3 in *Stuckenia*, which did not suggest an assignment of hybrids to independent cluster, but a split of *S. filiformis* consistent to genetic structure in Wu et al. (2020). For *Ranunculus*, most hybrids were F1s, while the hybrid individual in MQR was assigned to backcross-*R. subrigidus*. Additionally, three individuals with DJ were suggested to be F1s (Table 1 and Figure 4C).

One cpDNA fragment (*rpl32-trnL*) was sequenced for kinship identification. The lengths of the *rpl32-trnL* alignment region were 731 bp for *Stuckenia* and 887 bp for *Ranunculus*. For *Stuckenia*, 26 polymorphic sites were found between the parental species (Supplementary Figure 13). Twenty-one haplotypes (H1–H21) were found in *S. filiformis* populations, and three haplotypes (H22–H24) corresponded to *S. pectinata* (Supplementary Figure 13A). Among the hybrids suggested by SSR data, only individuals from GD had the haplotype of *S. pectinata*, whereas cpDNA haplotypes of other hybrids were assigned to *S. filiformis* (Table 1). For *Ranunculus*, 21 polymorphic sites were found between the parental species (Supplementary Figure 13B). Two and four haplotypes were found in *R. subrigidus* (S1–S2) and *R. trichophyllus* (T1–T4), respectively (Supplementary Figure 13B). The hybrids identified by microsatellite loci from DTR and MQR had haplotypes of *R. subrigidus*, while other hybrids contained the cpDNA of *R. trichophyllus* (Table 1).

Overall, combining the microsatellite and cpDNA data revealed the distributions of hybrids. Twenty-three individuals from eight sites were identified as hybrids of *Stuckenia* (Table 1). The hybrids were suggested to be post-F1s, and *S. filiformis* tended to be the maternal species (Table 1). Half of the mixed populations contained moderate proportions of hybrid individuals (Table 1). Thirty-one hybrids from nine sites were found in *Ranunculus*, and they were likewise not dominant in mixed populations except NM (Table 1). Most hybrid individuals of *Ranunculus* were F1s, while *R. trichophyllus* was more frequent, and reciprocal hybridization occurred in Qinghai, where *R. trichophyllus* was rarely distributed (Table 1 and Figure 1).

We assessed the distribution suitability of parents at the locations of their hybrids based on SDM results. We found that most sites of hybrids were suitable for at least one parental species, except that DL of *Myriophyllum*, HX and MQS of *Stuckenia*, NM and RT of *Ranunculus* were rarely suitable for either parent (Supplementary Table 2). According to the PC scores of the hybrid populations in “pca-env” analysis, many were situated in the overlapping niches of parents, while the DL of *Myriophyllum*, HX, GEM and MQS of *Stuckenia*, DTR, MQR, RT, and NM of *Ranunculus* were distributed in the marginal niches of both parents (Supplementary Figure 14 and Supplementary Table 2).

## Discussion

In the present study, we found extensive niche overlap in related aquatic taxa on highlands, according to the tests of niche similarity based on different approaches. This result suggested that highlands and adjacent regions provided suitable niches for contact with related aquatic species. The results showed expansive potential coexistence regions between all three species pairs on the QTP. We subsequently performed molecular analysis for the ambiguous populations and found that hybridization events were frequent in all species pairs. Although infrequent hybridization might be possible (Chung et al., 2005), broad overlap in geographic distribution should be optimal for the formation of hybrid zones or hybrid swarms (Yakimowski and Rieseberg, 2014). Therefore, extensive overlap of ecologically divergent aquatic plants across altitude gradients might greatly contribute to their frequent hybridization. Hybridization plays an important role in the spectacular radiation and diversification of plants on the QTP and its adjacent regions (Wen et al., 2014; Yang et al., 2019), which contributes to a high diversity of plant species, including a large number of endemics, within the region, especially the southeast edge (Wu, 1988; Xing and Ree, 2017; Wu et al., 2022). High aquatic plant diversity was also observed on the QTP, especially the southern and eastern QTP, and the species of interest were the most common water plants there (Wang, 2003). Based on the results of this work, we strongly advocate for further investigation on whether hybridization events are prevalent and important in the diversification of other taxa, particularly species in floating or emerged life form.

The eastern edge of the plateau and the Yarlung Zangbo River basin were the main overlapping areas for related aquatic species, which were characterized by great environmental gradients along altitude. The areas also accommodated a large proportion of aquatic hybrid populations. Compared to the plateau edge, the relatively warm areas of river drainage basins that provided broad wetland habitats were more important for the sympatry of related aquatic taxa (Wang, 2003; Abbott and Brennan, 2014). We found that the overlapping regions and hybrid populations were not restricted at middle altitudes but occupied a large altitude range, probably because of the intrazonal distribution of parental species (Santamaría, 2002). This might be decisive for the high niche overlap of related aquatic plants in highland areas. In these areas, hybrids were mostly advanced offspring, and hybrid superiority was widely detected in *Myriophyllum*, suggesting the potential to generate genetic novelty through introgression (Abbott and Brennan, 2014).

Hybridization in land plants on the QTP has been widely reported (Sun Y. et al., 2014; Jiang et al., 2016; Li et al., 2020; Wu et al., 2022) and is more likely to follow the scenario of allopatric divergence in alpine and second contact *via* postglacial expansion (Wen et al., 2014; Li et al., 2020). In contrast to the situation in land species, the interaction between related aquatic plants was not constrained to a few narrow joint zones but was found in several discrete overlapping regions. Western and northern Tibet, the Qajdam Basin, and some other valleys at high altitudes (e.g., the Hengduan Mountains), which were away from the main contiguous transition areas, provided additional junctures that connected feasible habitats for parental plants. These areas were patchily distributed and ecologically diverse and characterized by more rigorous conditions of temperature, aridity, salinity, and isolation (Shen et al., 2014). In these areas, a certain number of genetically mixed individuals and various hybridization patterns were detected in all species pairs. This implied that local contact and independent interaction between pure species might facilitate genetic adaptation across heterogeneous habitats (Yakimowski and Rieseberg, 2014; Harrison and Larson, 2016), especially under bounded or novel environments (Burke and Arnold, 2001; Gompert et al., 2017; Pierce et al., 2017).

We suggested the following factors that might affect the hybridization pattern by comparing the three parental species pairs. (1) The extent of niche overlap. Relatively higher niche overlap and hybridization frequencies were found in *Myriophyllum* and *Ranunculus* species. More opportunities for sympatry would create diverse genotypes to maintain environmental selection and in competition with the parents (Ortego et al., 2014). (2) The genetic incompatibility. Genetic novelties in post-F1s and further backcross hybrids might facilitate adaptative introgression into highlands in *Myriophyllum* (Burke and Arnold, 2001; Wu et al., 2015), while the genetic incompatibility between parental *Ranunculus* and *Stuckenia* species was more severe; (3) Phylogeographic history. Alpine endemic genetic clades of *S. filiformis* and *Ranunculus* species were dated to occur in the Pliocene and early Pleistocene (Chen J.M. et al., 2014; Du and Wang, 2016). However, the split between *M. sibiricum* and *M. spicatum* did not occur earlier than the Quaternary (Chen L. et al., 2014), and no specific genetic clade was identified on the plateau (Wu et al., 2016). This implied that *Myriophyllum* colonized the plateau recently and probably benefited from adaptation and founder effects in new habitats.

Hybridization in all studied species pairs was not reported until recently (Kaplan, 2008; Wiegleb et al., 2017), but few examples of *Stuckenia* and *Ranunculus* have been deeply investigated (Hollingsworth et al., 1996; Zalewska-Gałosz et al., 2015). Hybrids between *S. pectinata* and *S. filiformis* were described as *S.* x *suecicus* in North Europe (Hollingsworth et al., 1996), but investigation was limited in the diversity center of the genus, Center Asia and adjacent regions (Kaplan, 2008, but see Du and Wang, 2016). We found a scattered distribution of *S. pectinata* in the hinterland of the QTP, and hybridization between the two species was not rare in this region. Independent and frequent hybridization events and high variation in hybrids were suggested by both previous studies and our results (Kaplan, 2008; Du and Wang, 2016). Hybrids between *S. pectinata* and *S. filiformis* were predicted to be sterile (Hollingsworth et al., 1996), whereas we considered that the possibility of sexual events in hybrids or ancient intercrosses could not be rejected (Taylor and Larson, 2019), because many hybrids were suggested to be post F1s, according to the results of molecular methods. Extensive hybridization between *R. trichophyllus* and *R. subrigidus* was first revealed in an alpine ecosystem (Wiegleb et al., 2017). The sterility of their hybrids was suggested, which might be attributed to the different ploidy pattern of two species (Wiegleb et al., 2017). The present study supported the importance of hybridization in the speciation of the genus *Stuckenia* and *Ranunculus* section *Batrachium* (Kaplan, 2008; Zalewska-Gałosz et al., 2015; Wiegleb et al., 2017).

## Conclusion

In this study, we found obvious niche overlap between related aquatic species in the alpine environment, which was surmised to be an important cause of their hybridization. Hybrid populations are mainly located at the broad transition of parental species. The relatively warm drainage basins at high altitude and plateau edges were the high-potential regions for the sympatry and hybridization of closely related aquatic plants. These regions also accommodate high diversities of other aquatic taxa, and the influence of hybridization on diversification still needs additional empirical studies. The unique environment on the plateau also contributes to the hybridization of aquatic plants in discrete and novel habitats. We suggested that genetic compatibility, the extent of niche similarity and phylogeographic processes could influence hybridization patterns through comparisons among the three species pairs. Our findings highlighted that altitude gradients provided a suitable niche for extensive sympatry and the prevalence of hybridization in related aquatic species on the QTP. This result also implied the evolutionary importance of hybridization to the maintenance of aquatic plants in alpine environments. Genome-wide molecular markers are needed to finely evaluate the introgression patterns and evolutionary progress of aquatic taxa and to detect the corresponding adaptation mechanisms in future studies.

## Data Availability Statement

The datasets presented in this study can be found in online repositories. The names of the repository/repositories and accession number(s) can be found below: https://datadryad.org/stash, doi: 10.5061/dryad.gxd2547md, https://www.ncbi.nlm.nih.gov/genbank/, MT262969–MT262989, and MZ456343–MZ456351.

## Author Contributions

ZWu, XX, TL, and JiZ designed the study. ZWu, ZWa, JuZ, PC, and XL performed the field work and experiments. ZWu, ZWa, and DX analyzed the data. ZWu, XX, and TL wrote the manuscript. All authors contributed to the article and approved the submitted version.

## Conflict of Interest

The authors declare that the research was conducted in the absence of any commercial or financial relationships that could be construed as a potential conflict of interest. The reviewer SF declared a shared affiliation with several of the authors ZWa, JuZ, PC, XL, and XX to the handling editor at the time of review.

## Publisher’s Note

All claims expressed in this article are solely those of the authors and do not necessarily represent those of their affiliated organizations, or those of the publisher, the editors and the reviewers. Any product that may be evaluated in this article, or claim that may be made by its manufacturer, is not guaranteed or endorsed by the publisher.
