# Supplementary material for: Extensive Sympatry and Frequent Hybridization of Ecologically Divergent Aquatic Plants on the Qinghai-Tibetan Plateau
Source: Front Plant Sci. 2022 May 12;13:851151. doi: 10.3389/fpls.2022.851151 (PMC9135455; doi:10.3389/fpls.2022.851151)
Supplement: Supplementary file 1 [file Data_Sheet_1.docx]

**SUPPLEMENTARY MATERIAL**


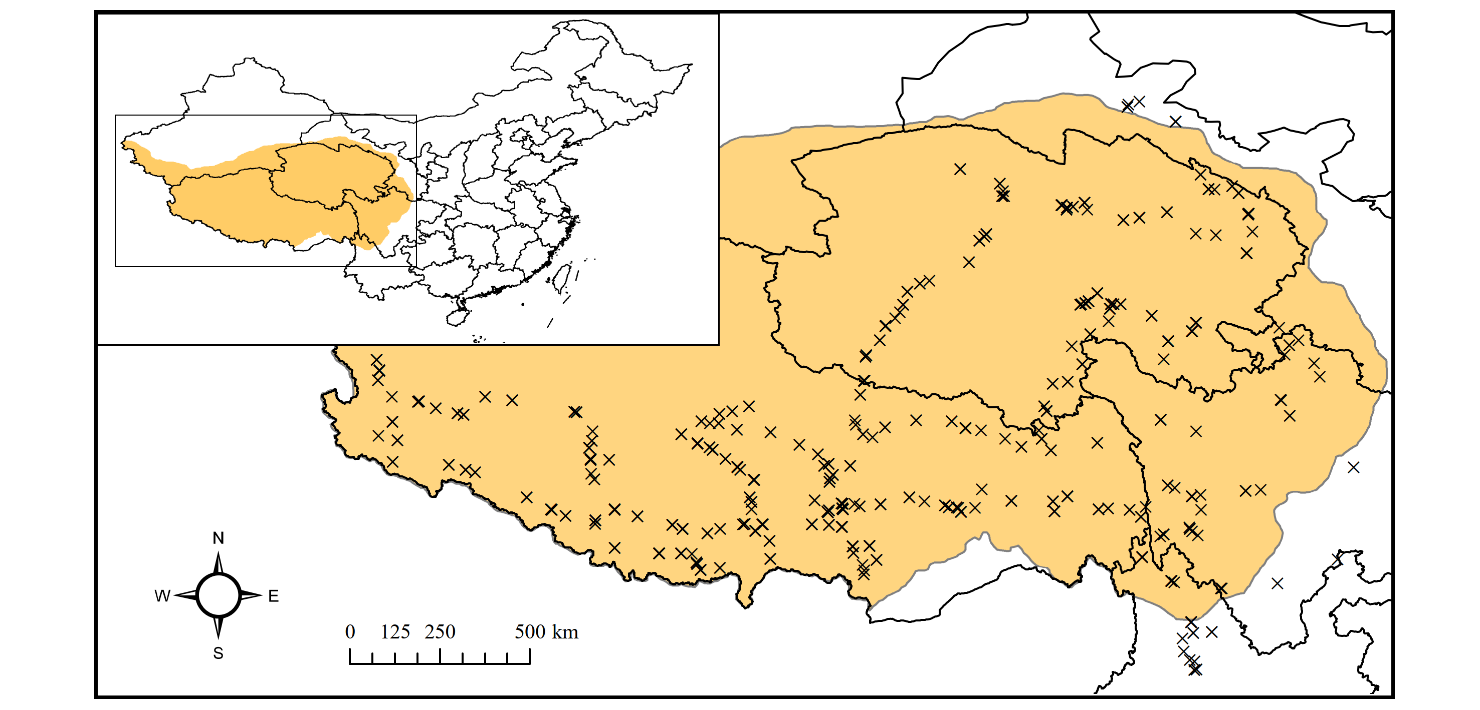


**Supplementary Figure 1** Geographic distribution of 254 survey sites on the Qinghai-Tibetan Plateau


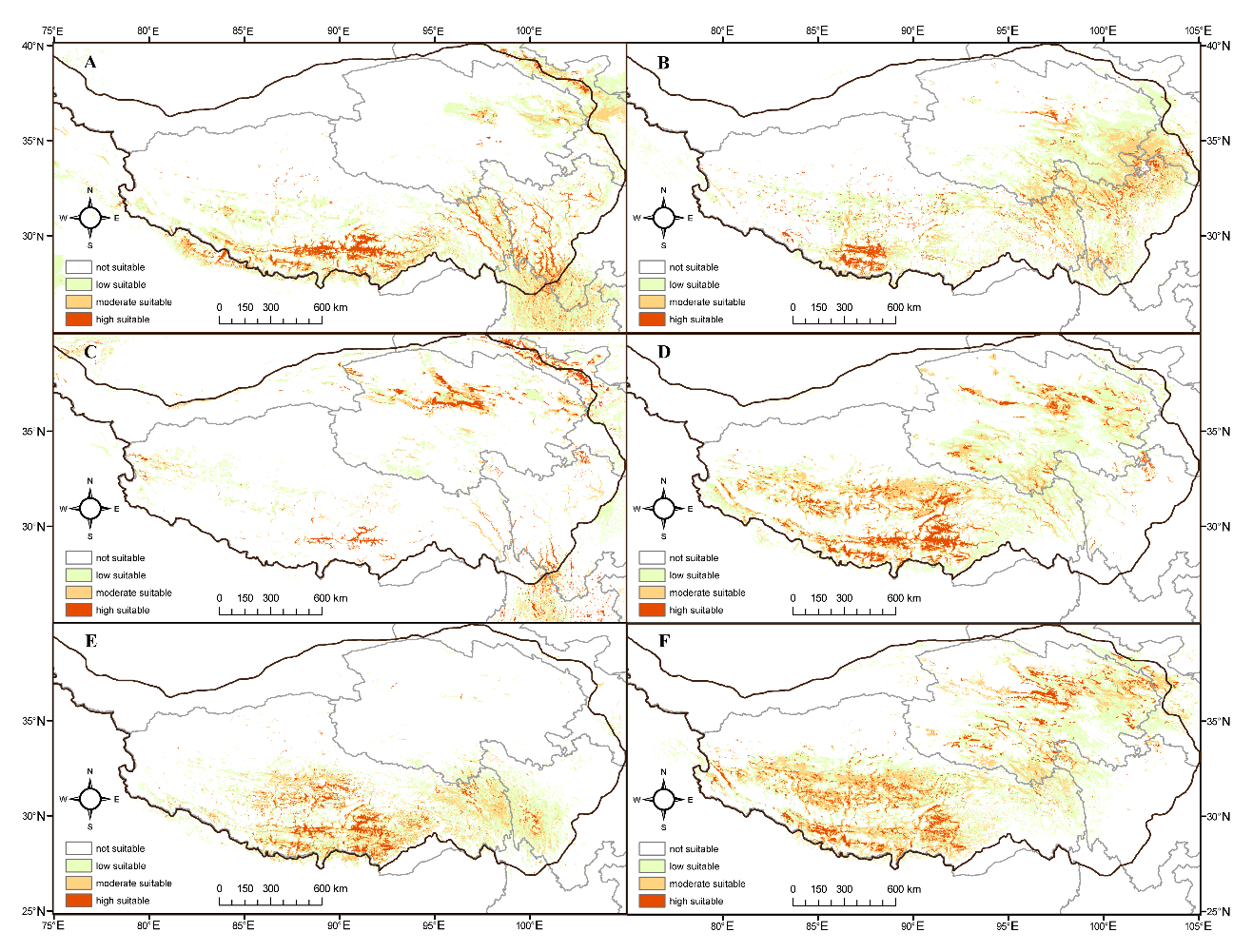


**Supplementary Figure 2** Species distribution models for the potential distributions of six parental species on the Qinghai-Tibetan Plateau. (A) *M. spicatum*, (B) *M. sibiricum*, (C) *S. pectinata*, (D) *S. filiformis*, (E) *R. trichophyllus*, and (F) *R. subrigidus*. The boundary of the QTP is shown.


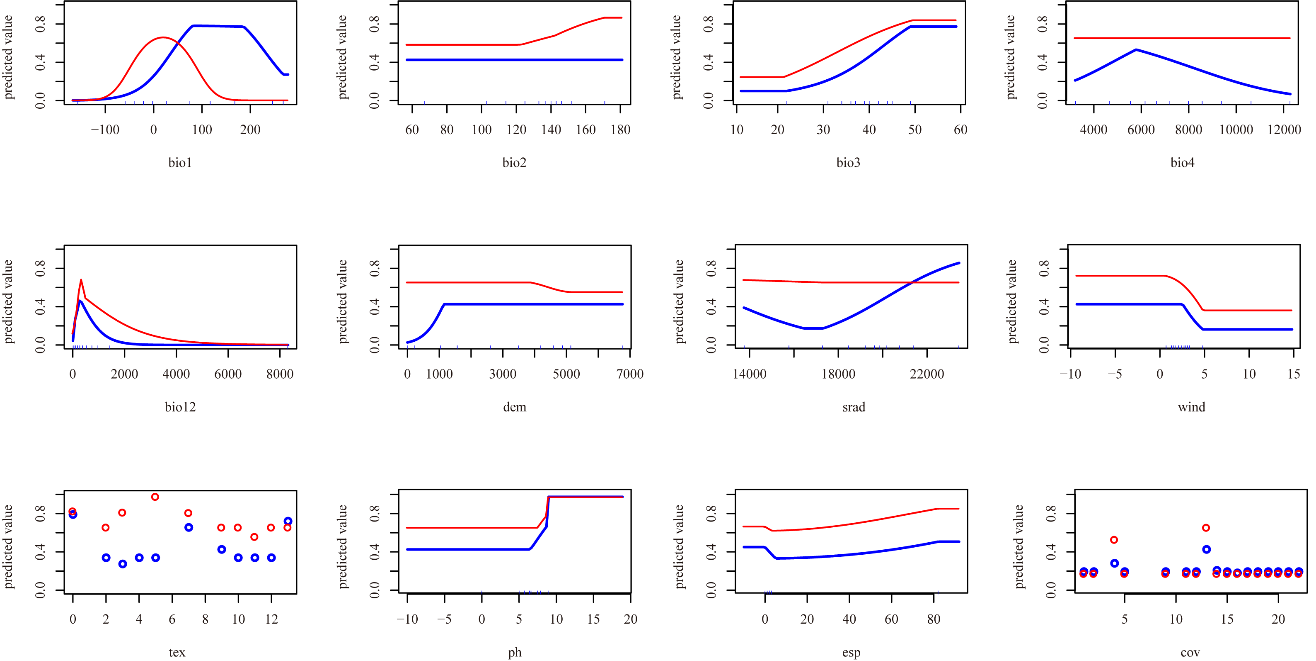


**Supplementary Figure 3** Response curves of each environmental variable for *Myriophyllum* in the Maxent models. Blue line/circle stands for *M. spicatum*, red line/circle stands for *M. sibiricum*; dem, elevation; srad, solar radiation; wind, wind speed; tex, topsoil USDA texture classification; ph, topsoil pH; esp, topsoil salinity; cov, landcover


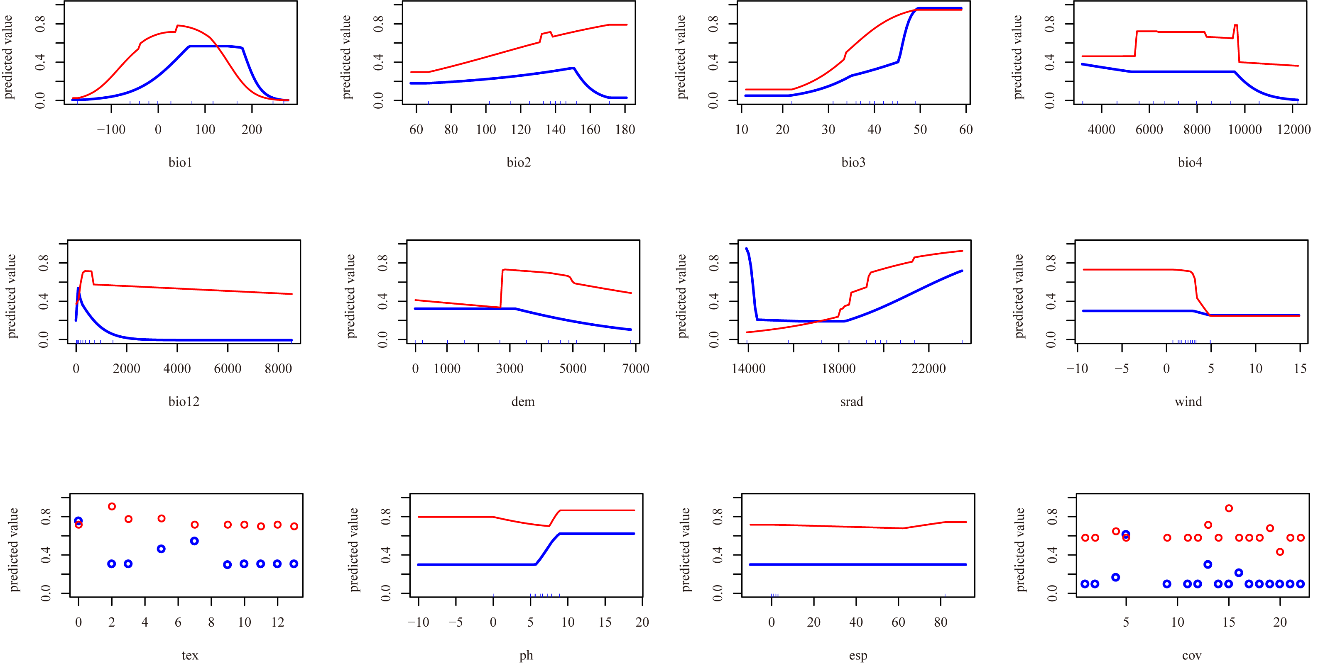


**Supplementary Figure 4** Response curves of each environmental variable for *Stuckenia* in the Maxent models. Blue line/circle stands for *S. pectinata*, red line/circle stands for *S. filiformis*; dem, elevation; srad, solar radiation; wind, wind speed; tex, topsoil USDA texture classification; ph, topsoil pH; esp, topsoil salinity; cov, landcover


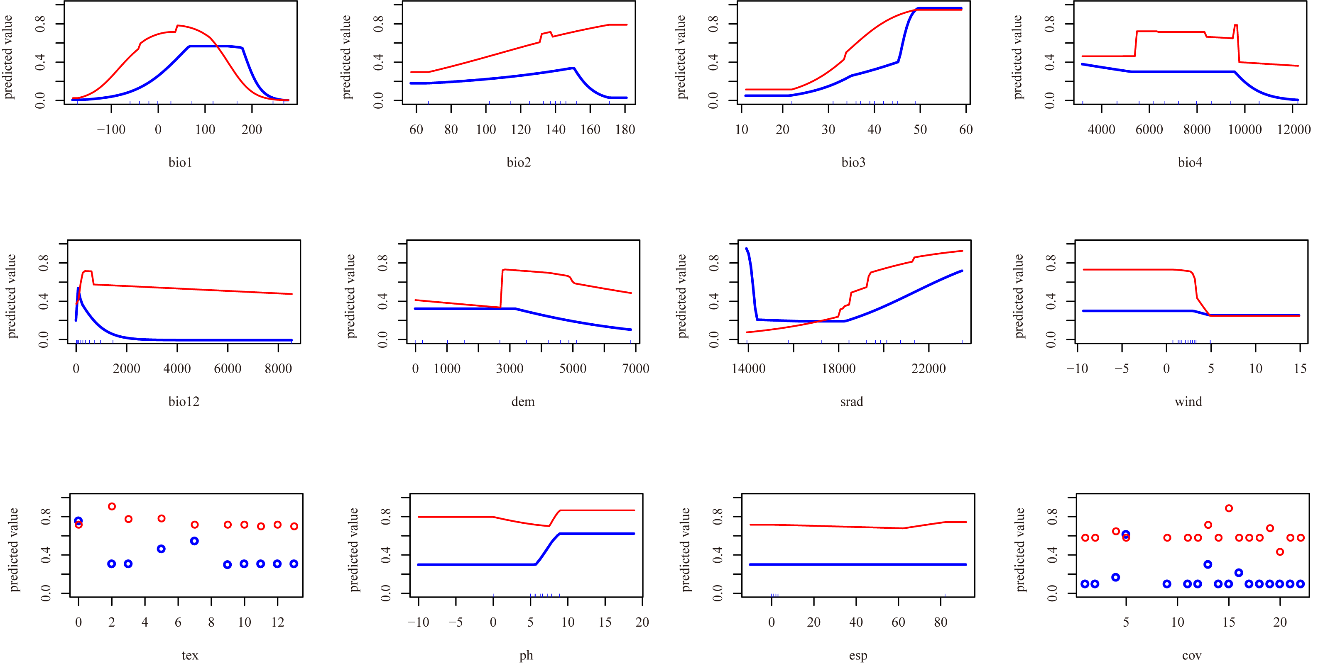


**Supplementary Figure 5** Response curves of each environmental variable for *Ranunculus* in the Maxent models. Blue line/circle stands for *R. trichophyllus*, red line/circle stands for *R. subrigidus*; dem, elevation; srad, solar radiation; wind, wind speed; tex, topsoil USDA texture classification; ph, topsoil pH; esp, topsoil salinity; cov, landcover

**
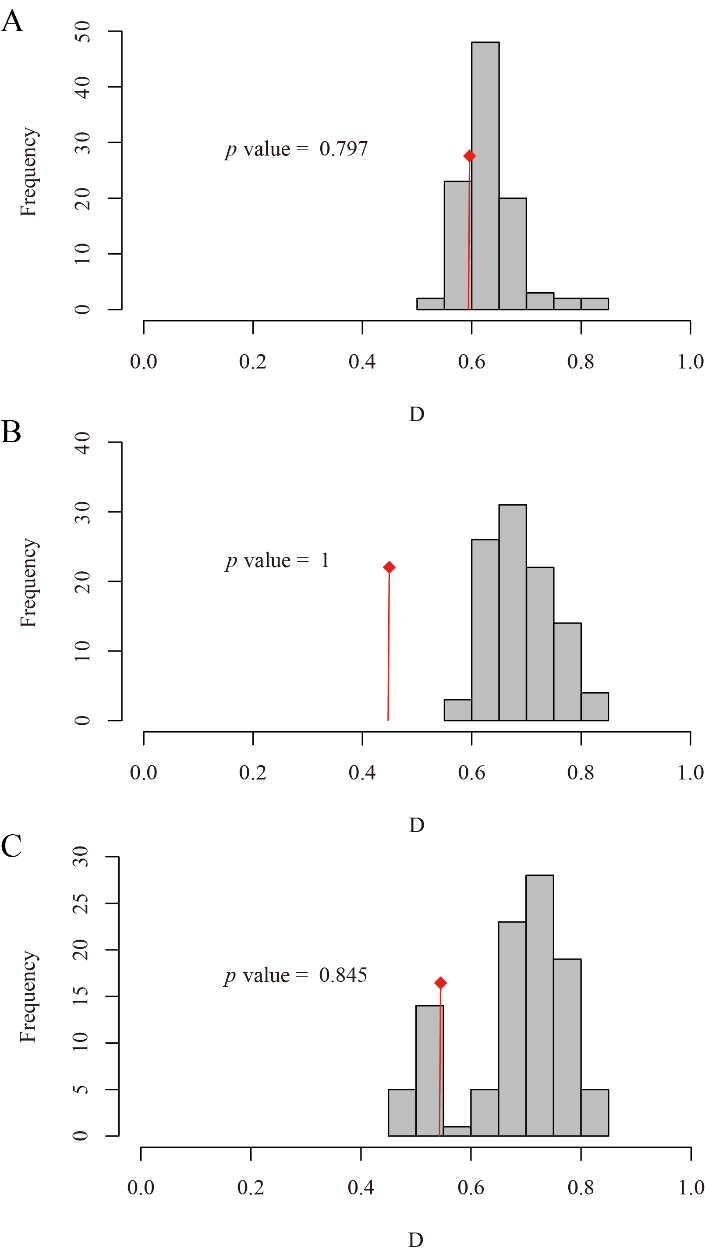
**

**Supplementary Figure 6** Results of niche equivalence tests based on species distribution models for comparison between widespread and boreal species. (A) *Myriophyllum*, (B) *Stuckenia* and (C) *Ranunculus*. Bars represent the observed niche overlap values, and histograms represent simulated niche overlaps by randomly reassigning the presence recodes to a pair of species


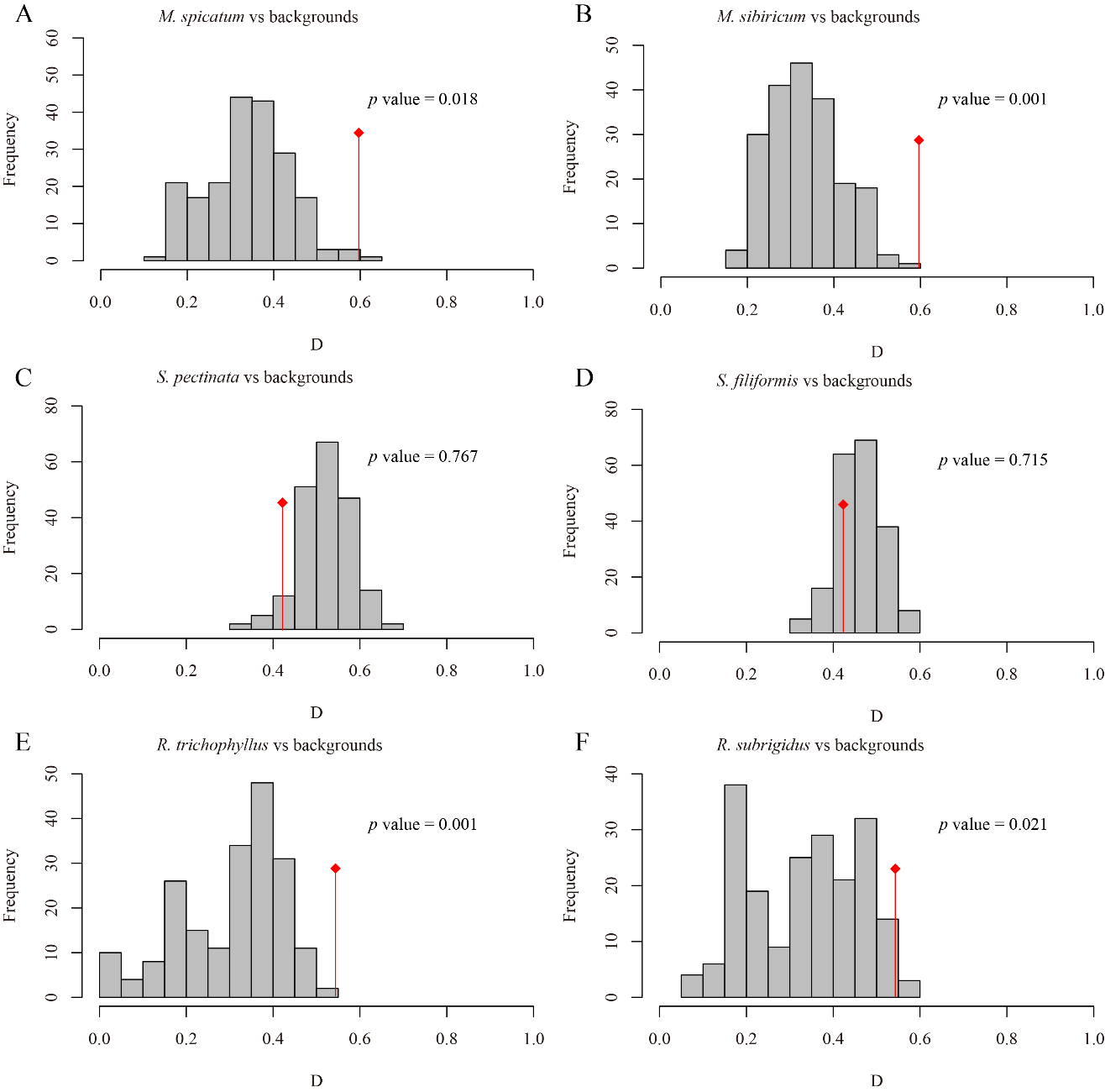


**Supplementary Figure 7** Results of background tests based on species distribution models. Bars represent the observed niche overlap between parental species, and histograms represent simulated niche overlaps between one parental species ((A) *M. spicatum*, (B) *M. sibiricum*, (C) *S. pectinata*, (D) *S. filiformis*, (E) *R. trichophyllus*, and (F) *R. subrigidus*) and the random backgrounds of the other.


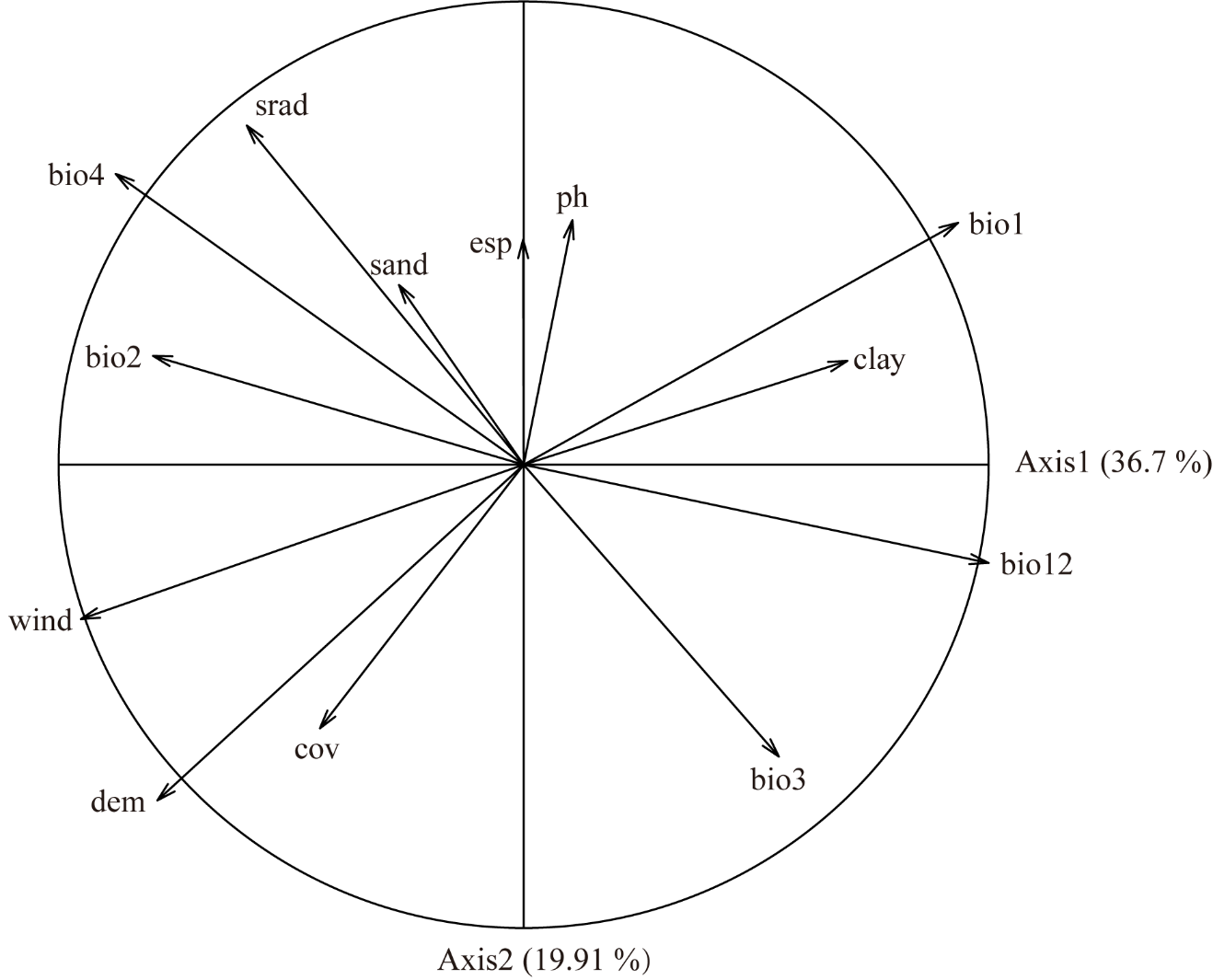


**Supplementary Figure 8** Contributions of the ﬁrst two PC axes to environmental space. Dem, elevation; srad, solar radiation; wind, wind speed; clay, proportion of clay in topsoil; sand, proportion of clay in topsoil; ph, topsoil pH; esp, topsoil salinity; cov, landcover


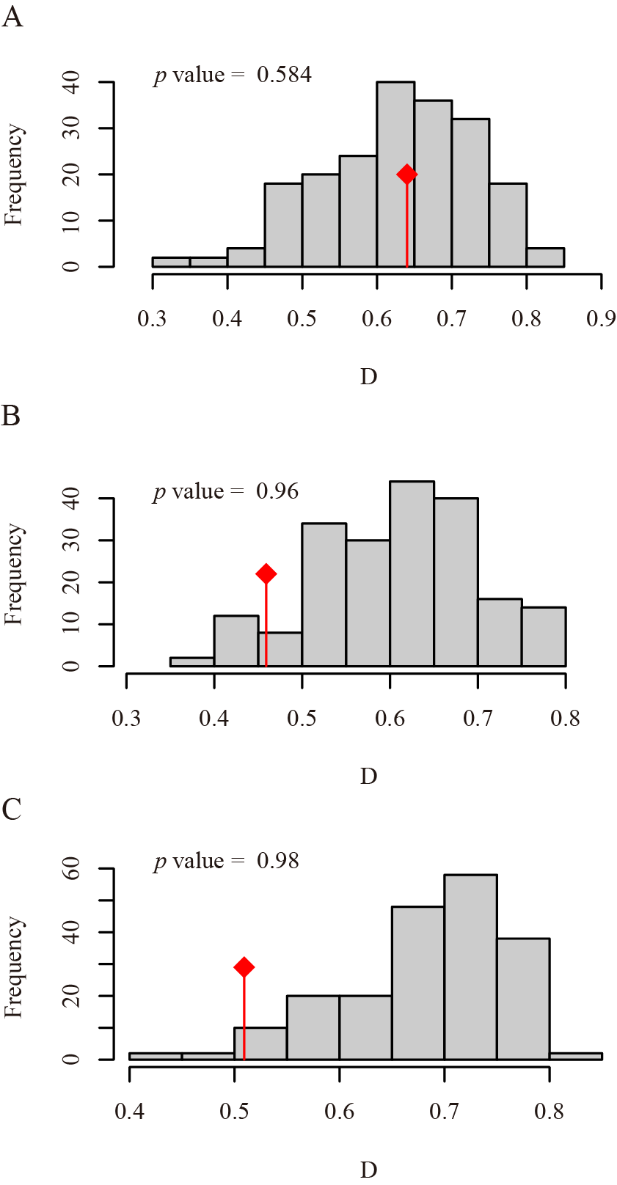


**Supplementary Figure 9** Results of niche equivalence tests based on pca-env technique between widespread and boreal species. (A) *Myriophyllum*, (B) *Stuckenia* and (C) *Ranunculus.* Bars represent the observed niche overlap values, and histograms represent simulated niche overlaps by randomly reassigning the presence recodes to a pair of species.


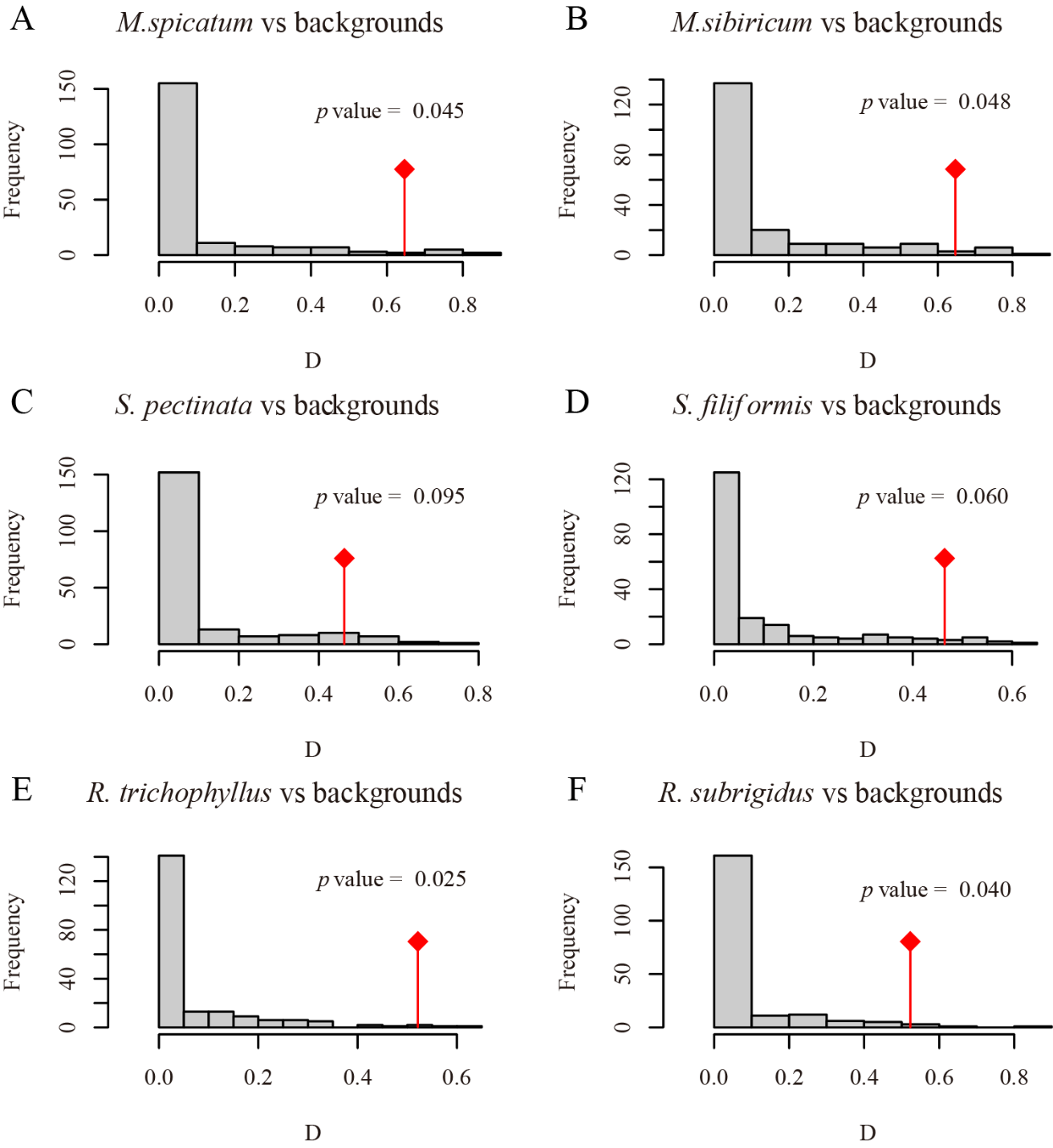


**Supplementary Figure 10** Results of background tests based on ‘pca-env’ technique. Bars represent the observed niche overlap between parental species, and histograms represent simulated niche overlaps between one parental species ((A) *M. spicatum*, (B) *M. sibiricum*, (C) *S. pectinata*, (D) *S. filiformis*, (E) *R. trichophyllus*, and (F) *R. subrigidus*) and the random backgrounds of the other.


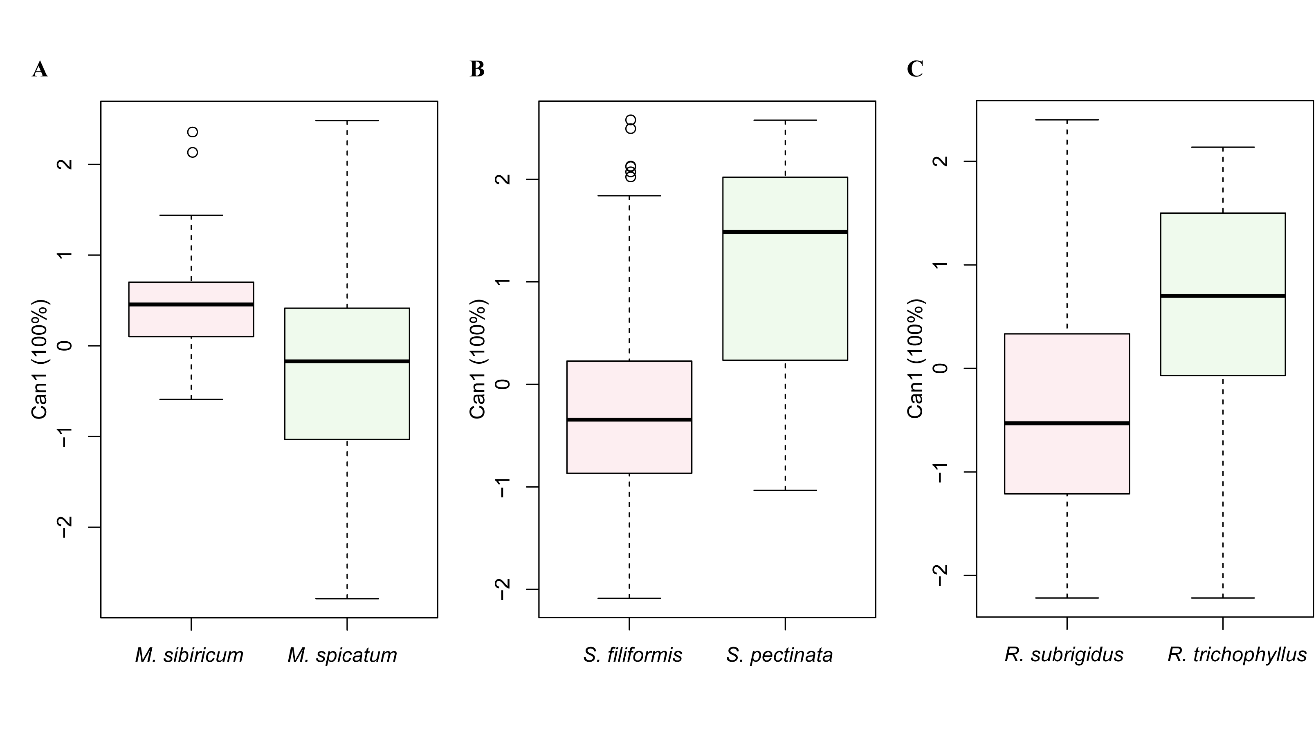


**Supplementary Figure 11** Canonical discriminant analysis (CDA) ordination of the three species pairs: (A) *Myriophyllum*, (B) *Stuckenia* and (C) *Ranunculus*.


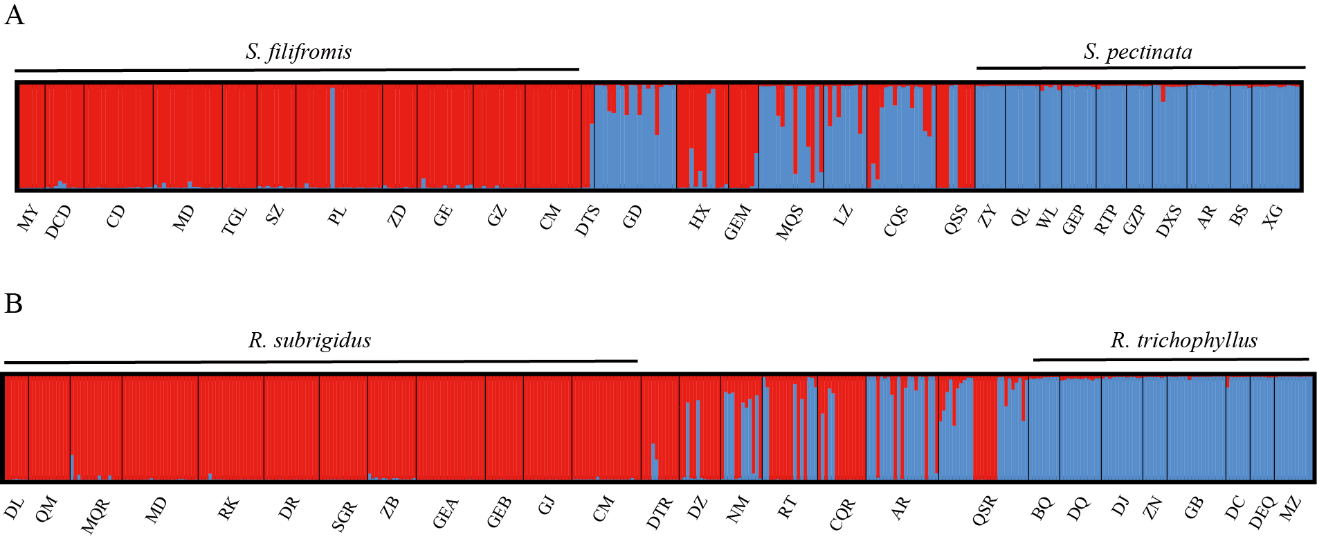


**Supplementary Figure 12** The results of STRUCTURE analysis for *Stuckenia* (A) and *Ranunculus* (B) based on microsatellite data. The vertical bars display the membership coefficient assigned to each parental species.


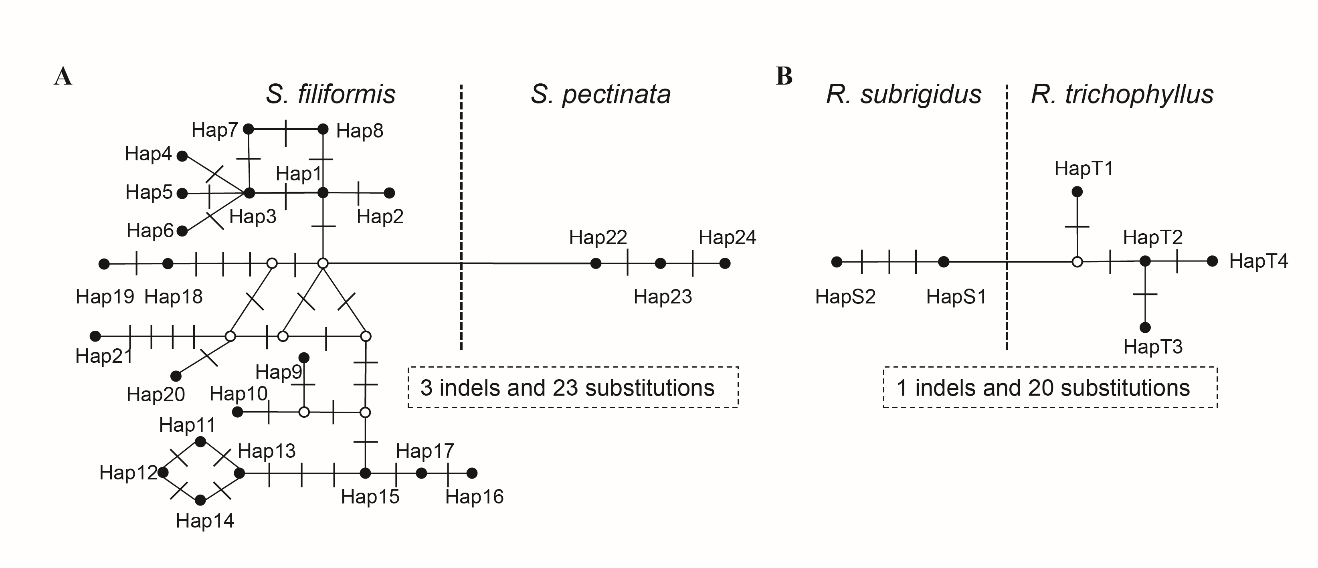


**Supplementary Figure 13** The network of haplotypes revealed in *Stuckenia* (A) and *Ranunculus* (B) based on a chloroplast fragment. Solid dots labeled with codes represent different haplotypes, and empty dots represent inferred interior nodes that were absent in the samples.


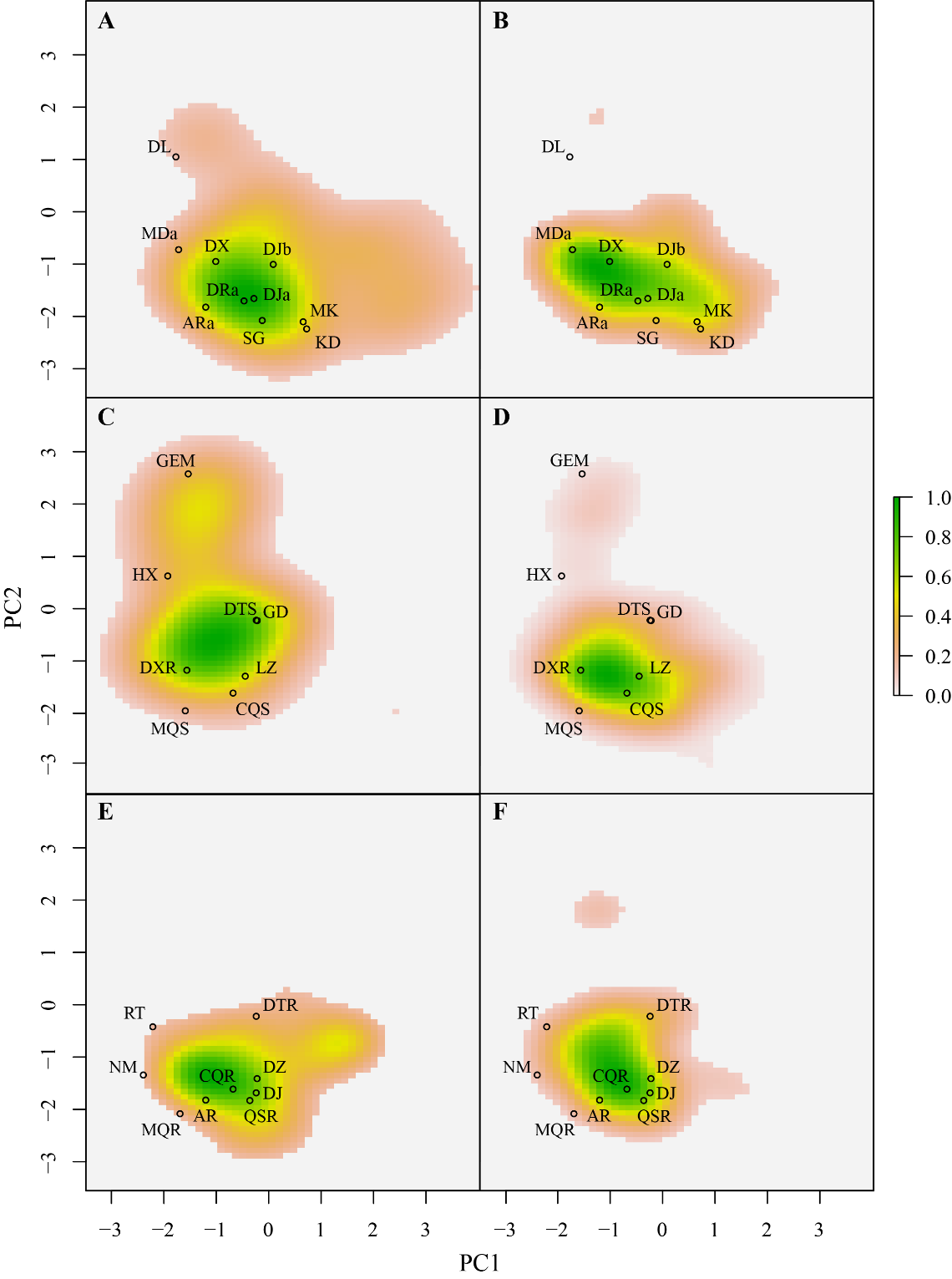


**Supplementary Figure 14** Niche comparison of hybrids with relative parental species, characterized by ‘pca-env’ technique. Colored areas represent the occurrence density for parental species, (A) *M. spicatum*, (B) *M. sibiricum*, (C) *S. pectinata*, (D) *S. filiformis*, (E) *R. trichophyllus*, and (F) *R. subrigidus*

**Supplementary Table 1** Area under the receiver operator curve (AUC) of training data and the threshold between presence and absence in the maxent models for each species

| Species | AUC | Threshold |
| --- | --- | --- |
| *M. spicatum* | 0.967 | 0.246 |
| *M. sibiricum* | 0.94 | 0.211 |
| *S. pectinata* | 0.934 | 0.259 |
| *S. filiformis* | 0.951 | 0.254 |
| *R. trichophyllus* | 0.954 | 0.231 |
| *R. subrigidus* | 0.945 | 0.276 |

**Supplementary Table 2** Suitability of parental species at the locations of hybrid populations

| Species | Code | PW | PB | DW | DB |
| --- | --- | --- | --- | --- | --- |
| *Myriophyllum* | SG | 0.732 | 0.464 | 0.778 | 0.405 |
| *Myriophyllum* | ARb | **0.092** | 0.362 | 0.638 | 0.583 |
| *Myriophyllum* | DRa | 0.784 | 0.685 | 0.984 | 0.737 |
| *Myriophyllum* | DJa | 0.695 | 0.607 | 0.979 | 0.700 |
| *Myriophyllum* | DJb | 0.493 | 0.472 | 0.632 | 0.578 |
| *Myriophyllum* | DX | 0.391 | 0.552 | 0.656 | 0.928 |
| *Myriophyllum* | MK | 0.532 | 0.481 | 0.516 | 0.503 |
| *Myriophyllum* | MDa | **0.013** | 0.369 | 0.287 | 0.707 |
| *Myriophyllum* | DL | **0.006** | **0.058** | 0.134 | 0.000 |
| *Myriophyllum* | KD | 0.437 | 0.436 | 0.396 | 0.376 |
| *Stuckenia* | DTS | 0.694 | **0.089** | 0.739 | 0.231 |
| *Stuckenia* | GD | 0.731 | **0.140** | 0.739 | 0.231 |
| *Stuckenia* | HX | **0.257** | **0.142** | 0.332 | 0.038 |
| *Stuckenia* | GEM | 0.299 | 0.276 | 0.311 | 0.046 |
| *Stuckenia* | MQS | **0.107** | **0.129** | 0.178 | 0.228 |
| *Stuckenia* | CQS | 0.399 | 0.668 | 0.424 | 0.813 |
| *Stuckenia* | LZ | 0.316 | 0.720 | 0.526 | 0.713 |
| *Stuckenia* | DXR | 0.428 | 0.636 | 0.761 | 0.852 |
| *Ranunculus* | DTR | **0.147** | 0.338 | 0.246 | 0.356 |
| *Ranunculus* | MQR | **0.176** | 0.353 | 0.235 | 0.188 |
| *Ranunculus* | RT | **0.087** | **0.141** | 0.156 | 0.218 |
| *Ranunculus* | CQR | 0.687 | 0.596 | 0.890 | 0.954 |
| *Ranunculus* | AR | 0.458 | 0.389 | 0.721 | 0.682 |
| *Ranunculus* | NM | **0.207** | **0.214** | 0.084 | 0.151 |
| *Ranunculus* | DJ | 0.627 | 0.722 | 0.717 | 0.686 |
| *Ranunculus* | DZ | 0.896 | 0.794 | 0.695 | 0.631 |
| *Ranunculus* | QSR | 0.706 | 0.529 | 0.691 | 0.685 |

PW, occurrence Probability of the Widespread parental species in Maxent models; PB, occurrence Probability of the Boreal parental species in Maxent models; DW, occurrence Density of the Widespread parental species using ‘pca-env’ approach; DB, occurrence Density of the Boreal parental species using ‘pca-env’ approach. PW and PB are in bold when lower than thresholds suggested by TSS.
